# The genetic association between major depressive disorder and coronary heart disease

**DOI:** 10.1017/neu.2024.40

**Published:** 2025-03-21

**Authors:** Yue Shi, Feikang Xu, Yumei Wei, Duan Zeng, Shen He, Jingjing Huang, Huafang Li

**Affiliations:** 1 Department of Psychiatry, Shanghai Mental Health Center, Shanghai Jiao Tong University School of Medicine, Shanghai, China; 2 Schizophrenia Program, Shanghai Mental Health Center, Shanghai Jiao Tong University School of Medicine, Shanghai, China; 3 Shanghai Key Laboratory of Psychotic Disorders, Shanghai Mental Health Center, Shanghai Jiao Tong University School of Medicine, Shanghai, China; 4 Shanghai Clinical Research Center for Mental Health, Shanghai, China

**Keywords:** major depressive disorder, coronary heart disease, genetics

## Abstract

Major depressive disorder (MDD) and coronary heart disease (CHD) can both cause significant morbidity and mortality. The association of MDD and CHD has long been identified, but the mechanisms still require further investigation. Seven mRNA microarray datasets containing samples from patients with MDD and CHD were downloaded from Gene Expression Omnibus. Combined matrixes of MDD and CAD were constructed for subsequent analysis. Differentially expressed genes (DEGs) were identified. Functional enrichment analyses based on shared DEGs were conducted to identify pivotal pathways. A protein-protein network was also applied to further investigate the functional interaction. Results showed that 24 overlapping genes were identified. Enrichment analysis indicated that the shared genes are mainly associated with immune function and ribosome biogenesis. The functional interactions of shared genes were also demonstrated by PPI network analysis. In addition, three hub genes including MMP9, S100A8, and RETN were identified. Our results indicate that MDD and CHD have a genetic association. Genes relevant to immune function, especially IL-17 signalling pathway may be involved in the pathogenesis of MDD and CHD.

## Significant Outcomes

1. Genetic association between major depressive disorder and coronary heart disease was reported in this study.
2. Three hub genes including MMP9, S100A8, and RETN were identified in the overlapping DEGs of MDD and CHD.
3. Immune function and ribosome biogenesis are pivotal pathways connecting MDD and CHD.

## Limitations

1. This study lacks experimental validation.
2. Comparation of different methods of combined matrixes construction may help better data mining.

## Introduction

Major depressive disorder (MDD) and coronary heart disease (CHD) are both important diseases leading to significant health burdens. MDD is now the number one single contributor to disability in the world according to the WHO’s 2017 Health Report (Benziger *et al*., 2016). More than 300 million people suffer from MDD, which is about 4.4 percent of the global population, and the incidence is twice as high in women as in men (Salk *et al*., 2017, Parker and Brotchie, 2010). MDD is also the leading cause of death by suicide (Hawton *et al*., 2013), accounting for as many as 800,000 people each year. Therefore, it’s crucial to further investigate the aetiology and pathogenesis of this debilitating mental disorder.

CHD, also known as coronary artery disease (CAD), is also among the leading causes of mortality, accounting for 7.4 million deaths worldwide (De Hert *et al*., 2018). Its characteristic lesion is the accumulation of fatty and/or a fibrous substance (Libby *et al*., 2019), also called atherosclerotic plaques, in the arteries of the heart, which can interrupt or block the heart’s coronary circulation and lead to tissue ischemia. CHD can cause angina pectoris, myocardial infarction, and chronic ischemia (Doenst *et al*., 2019). In the case of severely stenotic CHD, myocardial infarction or chronic ischemia may cause heart failure and/or even death (Doenst *et al*., 2019).

There is an association between MDD and CHD. For example, the symptom of chest tightness, palpitations, low energy, disturbance of sleep, and difficulty in carrying out daily routines can be observed in both patients with MDD and patients with heart diseases (Howard *et al*., 2019, Malhi and Mann, 2018). The comorbidity of the two diseases is quite common, with a prevalence rate of MDD being about 10 to 31% in patients with CHD (Vaccarino *et al*., 2020). This was far higher than the prevalence rate of 4.4% in the global population estimated by WHO. What’s more, several meta-analyses have shown that patients with baseline MDD symptoms were associated with increased risk (Harshfield *et al*., 2020, Rajan *et al*., 2020, Correll *et al*., 2017), poor prognosis (Lichtman *et al*., 2014), and lower quality of life (O’Neil *et al*., 2013) for CHD. Such association leads to the development of psycho-cardiology, a field that investigates the role of psychosocial factors in the emergence, the course and the rehabilitation of cardiac diseases. However, the mechanisms underlying such a relationship remain unknown despite the increasing academic attention. One possible factor is that the changes in the autonomic nervous system and neurohormonal function. Studies have shown that there is a shift in the autonomic balance in depressed patients, manifested by the leaner relationship between heart rate variability (HRV) reduction and MDD severity (Kemp *et al*., 2010). And in patients with a history of CHD, increased sympathetic activity and reduced HRV (Stapelberg *et al*., 2012) are also commonly observed. Patients with MDD also show higher levels of plasma and urinary catecholamines (Carney and Freedland, 2017). Other potential biological mechanisms include platelet dysfunction (Carney and Freedland, 2017), endothelial dysfunction, such as plasminogen activator inhibitor-1 and fibrinogen (Hare *et al*., 2014), and abnormalities in the inflammatory response (Carney and Freedland, 2017, Wu *et al*., 2021). Behavioural mechanisms are also factors that potentially link MDD and CHD. Patients with comorbid CHD and MDD are prone to develop unhealthy behaviours and lifestyles, for example, smoking, sedentary lifestyle, poor adherence to medication, and poor dietary habits (Carney and Freedland, 2017). Newly raised possibilities are pointing towards gut microbiota and endocrine signalling. Although CHD and MDD are both polygenic and multifactorial disorders, the genetic association between these two diseases is less studied. Kenneth et al., found in 30,374 twins that the comorbidity in women arose primarily from genetic effects( + 0.16) (Kendler *et al*., 2009), while genetic effects played a greater role in younger male patients. Gloria and her colleagues (Li *et al*., 2020) evaluated the bidirectional causal association using Mendelian randomisation and demonstrated that both MDD phenotypes were genetically correlated with cardiovascular diseases. Yunlong Lu et al. (Lu *et al*., 2021) later performed Mendelian randomisation estimates on 807553 individuals with MDD and 60,801 cases of CAD, and results showed that genetic liability to MDD is associated with an increased risk of CHD. However, whether there are overlapping related genes and pathways have not been further investigated.

In this study, we conducted a bioinformatics analysis to explore the genetic overlap of MDD and CAD. Differentially expressed genes (DEGs) identified in both disorders were collected to perform functional analyses. Weighted gene co-expression network analysis (WGCNA) was also performed in both MDD and CAD to identify key module genes.

## Method

### Data sources

This analysis was based on mRNA microarray datasets downloaded from the Gene Expression Omnibus (GEO, http://www.ncbi.nlm.nih.gov/geo/), a database of the National Center for Biotechnology Information (NCBI). All relevant series publicly available up to June 2024 on GEO were searched. MDD datasets were searched using ‘MDD’, ‘depression’ or ‘major depressive disorder’. CAD datasets were searched using ‘CAD’, ‘coronary heart disease’ or ‘coronary artery disease’. Sample type is limited to peripheral blood and the sample size must be greater than 20. Only data series that both patients and healthy control are available in one single dataset were included. In order to reduce the interference of confounding factors, patient samples or data sets used as comorbidity studies other than MDD or CAD in the original study were excluded. In addition, CAD cases are defined as patients with ≥70% stenosis in >1 major vessel or ≥50% stenosis in >2 arteries, or patients with Duke CAD index (CADi) >23. To follow-up studies of treatment, only baseline information was retained. Finally, seven mRNA datasets were selected: MDD studies include GSE98793, GSE52790, GSE19738, and GSE201332. CAD studies include GSE10195, GSE12288, and GSE20680 (Table 1). Written informed consents were obtained from all participants in original studies, and the studies were approved by ethic committees. Trials of the datasets were approved by local institutional review boards, and informed consents were provided by all subjects according to original articles.

**Table 1.** MDD and CAD expression profile data sets from GEO database

			Number					
Database (MDD/CAD)	Dataset ID	Platform	**MDD/CAD**	**Control**	Type	Tissue	Age (Year)	Gender (Male/Female)
MDD	GSE98793	GPL570	128	64	mRNA	Whole blood	52.0 ± 11.45	48/144
MDD	GSE52790	GPL17976	10	12	mRNA	Whole blood	NA	11/11
MDD	GSE19738	GPL6848	33	34	mRNA	Whole blood	42.5 ± 12.02	26/41
MDD	GSE201332	GPL19072	20	20	mRNA	Whole blood	38.3 ± 10.76	20/20
CAD	GSE10195	GPL1708	27	14	mRNA	Whole blood	NA	NA
CAD	GSE12288	GPL96	110	112	mRNA	Whole blood	53.3 ± 7.36	172/50
CAD	GSE20680	GPL4133	87	52	mRNA	Whole blood	NA	NA

GEO, Gene Expression Omnibus; MDD, major depressive disorder; CAD, coronary artery disease.

### Dataset preparation

Publicly available data were downloaded from GEO using the GEOquery R package. Probe ids were converted to official gene symbols using platform annotation files available. Gene expression information was obtained from series of matrix TXT files except GSE10195. The matrix provided by this dataset contains a large number of negative numbers. Therefore, raw data of GSE10195 were downloaded and background corrected to construct a normalised gene expression matrix. When all gene expression matrixes and clinical information (disease or healthy control) were ready, the four datasets from four MDD studies and the three datasets from three CAD studies were combined separately to obtain two combined matrixes. Batch effects (Sui *et al*., 2023), which refer to potential differences in the data from different sources caused by non-biological factors, for example, different processing time or staff, were eliminated using function ComBat from sva R package (Figure. S1–S2). The code is as follows:

*model=model.matrix(∼as.factor(Group))*

*combat_edata=ComBat(dat = as.matrix(dat),*

*batch = batch,*

*mod = model,*

*par.prior=TRUE,*

*ref.batch = 1)*

### Identification of differentially expressed genes

The limma package in R studio was used to process the significance analysis of DEGs between patients and healthy controls of MDD and CHD, separately. We employed the default Benjamini–Horchberg method to calculate adjusted p-values (Geng and Huang, 2021; Hu *et al*., 2021; Long *et al*., 2021). In both combined datasets, genes with adjusted *p* value <0.05 and |log2 (foldchange)|> 0.1 as the cut-off criterion were considered as DEGs. Shared DEGs between MDD and CHD were selected for subsequent analysis.

### Functional enrichment for shared DEGs

The Gene Ontology (GO) (Consortium, 2004) project provides a systematic description of biology, including consistent descriptors for gene products and standard classifications of sequences and sequence features, which has been widely used in annotation projects and biological analyses. Kyoto encyclopedia of genes and genomes (KEGG) (Kanehisa and Goto, 2000) is another knowledge base aimed to link individual genes with higher functional annotation and regulatory pathways. For a more extensive understanding of the DEGs, we performed GO (including biological process, molecular function, and cellular component) and KEGG enrichment analyses using clusterProfiler R package (Yu *et al*., 2012) and Metascape (Zhou *et al*., 2019). GO terms and KEGG pathways with *P* value <0.05 were considered statistically significant. Then we used the ggplot2 and GOplot R package to visualise the results.

### PPI network construction and hub gene analyses

To further investigate the functional interaction network of DEG-encoding proteins in both MDD and CHD, Search Tool for the Retrieval Interacting Genes (Szklarczyk *et al*., 2021) (STRING, https://string-db.org), a comprehensive database designed to integrate both physical interactions and functional associations between proteins, was applied to automatically predict and visualise the PPI network and clusters. The interaction score cut-off of 0.400 was set as a default option. Results were imported into Cytoscape (version 3.8.2) for hub gene analyses and visualisation. CytoHubba plugin and CytoNCA plugin were applied to identify the most connected genes as hub genes.

### WGCNA module identification

WGCNA R package is used to reconstruct the co-expression network and yield relevant modules. We applied the algorithm of WGCNA to both combined matrixes. A minimum module size of 30 and a soft threshold of R^2^ >0.9 were selected. The Pearson’s correlation coefficients were computed to describe the relationship between the samples and within modules. The cut-off criteria of absolute eigengene-based connective (kME) were set to be over 0.8.

## Results

### Identification of DEGs

**Figure 1.**
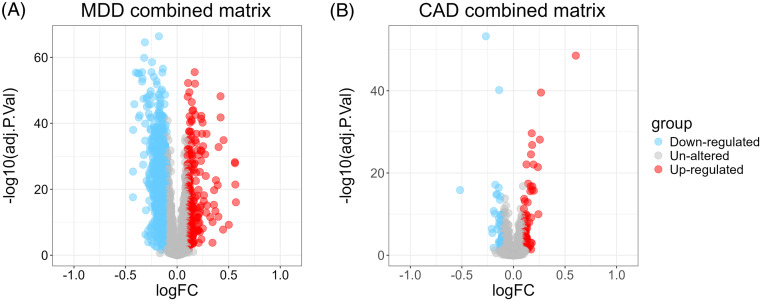
Volcano plot of combined matrix A. Volcano plot shows the differently expressed genes (DEGs) of combined major depressive disorder matrix. B. Volcano plot shows the DEGs of combined coronary artery disease matrix. MDD, major depressive disorder. CAD, coronary artery disease; FC, fold change.

**Figure 2.**
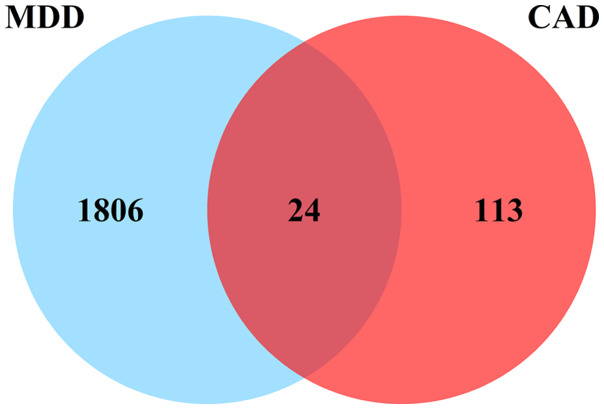
Venn diagram showing the number of overlapped genes.

A total of 1830 DEGs (Figure 1A) were identified in the MDD group compared to the control group in the combined MDD matrix, including 244 up-regulated DEGs and 1586 down-regulated DEGs. In the combined CAD datasets, 137 DEGs (Figure 1B) were identified from CHD samples and control samples, with 84 up-regulated and 53 down-regulated. There was a total of 24 overlapping DEGs (Figure 2) between MDD and CHD.

### Functional enrichment analysis for the overlapping DEGs

**Figure 3.**
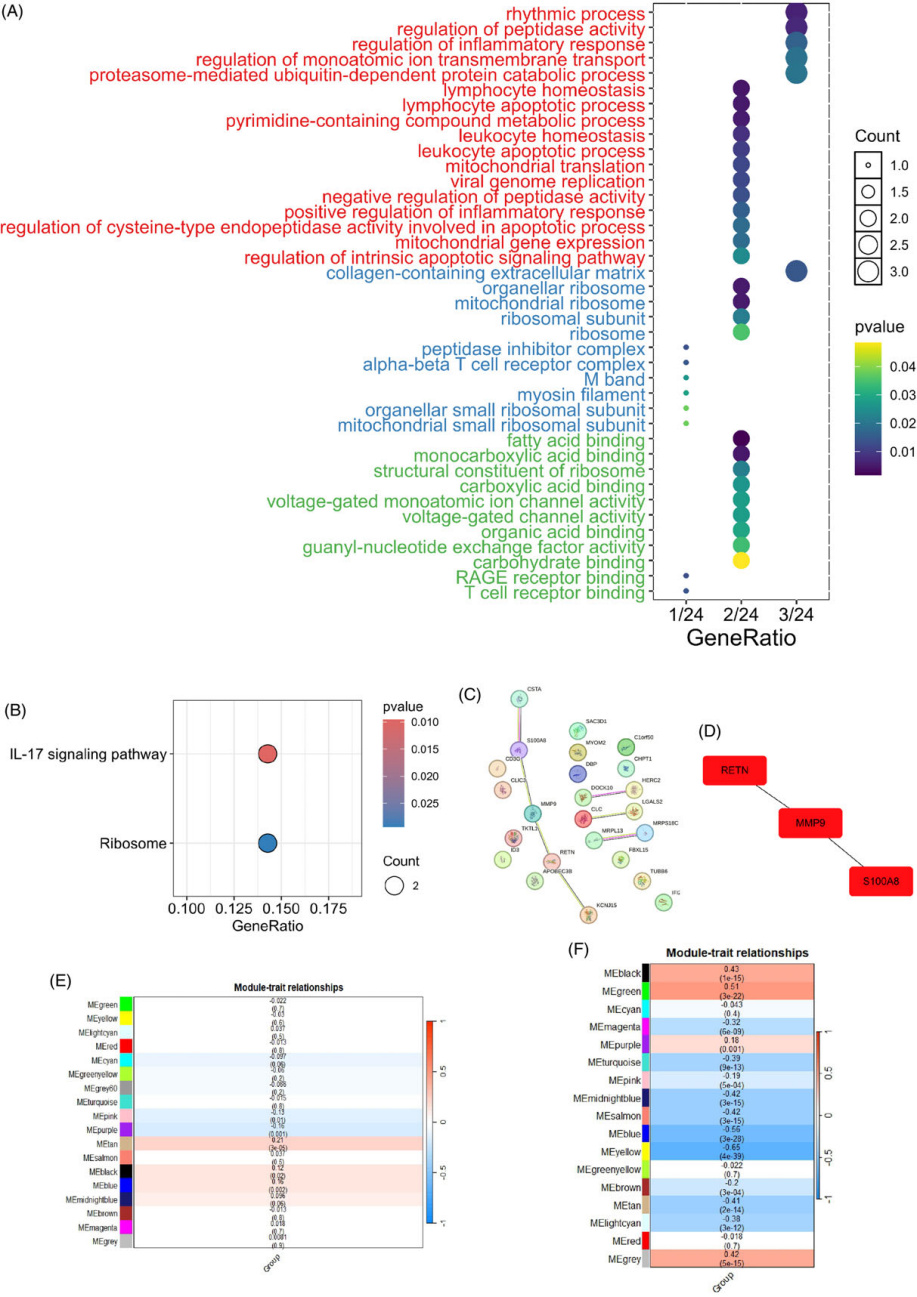
A. GO enrichment analysis base on overlapping genes of major depressive disorder (MDD) and coronary heart disease (CHD). B. KEGG pathway analysis based on overlapping genes between MDD and CHD. C. PPI network. D. Top three hub genes identified by Cytoscape. E. Correlation between modules and groups of CHD. F. Correlation between modules and groups of MDD.

GO and KEGG enrichment analyses were performed for the 24 shared DEGs between MDD and CHD. Results from clusterProfiler showed that in biological process, shared DEGs were mainly associated with rhythmic process, regulation of peptidase activity, lymphocyte and leucocyte homoeostasis and apoptotic process, mitochondrial translation, and protein catabolic process. (Figure 3A). Cellular component analysis indicated that overlapping DEGs were mainly enriched in collagen-containing extracellular matrix, organellar ribosome, mitochondrial ribosome, peptidase inhibitor complex and alpha-beta T cell receptor complex (Figure 3A). The major molecular function identified for these genes were fatty acid binding, monocarboxylic acid binding, structural constituent of ribosome, voltage-gated channel activity, guanyl-nucleotide exchange factor activity, RAGE receptor binding, and T cell receptor binding (Figure 3A). Moreover, significantly enriched KEGG pathways of overlapping DEGs were IL-17 signalling pathway and ribosome (Figure 3B). Enrichment results of Metascape also showed that the major pathways were NABA matrisome associated, adaptive immune system, and leucocyte differentiation (Figure.S3).

### PPI network analysis and hub gene analysis

Based on STRING database, a total of 11 proteins and 17 potential interactions were identified for 24 overlapping DEGs (Figure 3C), with PPI enrichment *p*-value <0.05 (*p* = 0.049). Results from STRING database were imported into Cytoscape for further analysis. According to results from CytoHubba plugin and CytoNCA, the top three hub genes of the highest degree of connectivity were MMP9, RETN, and S100A8, as shown in Figure 3D and Table.S1. Both MMP9 and S100A8 belong to the IL-17 signalling pathway (Figure.S4) while RETN belongs to cytokines and neuropeptides. MMP9 was up-regulated in both MDD and CAD (Figure.S5–S6). RETN was up-regulated in MDD but down-regulated in CAD, while S100A8 was down-regulated in MDD but up-regulated in CAD. In addition, the mRNA level of other key genes belonging to IL-17 signalling pathway also changed. For example, in the combined CAD matrix, the expression level of S100A9 increased. According to the combined MDD matrix, the expression level of MUC5 increased, while the expression level of COX2, NFKB1, APC5, A20, and Hsp90 decreased.

### WGCNA analysis show more genes and pathways associated with MDD and CAD

To further investigate the relationship between genes and the two diseases, we applied WGCNA algorithm to both combined matrixes. Results showed that, in CAD, MEpink, MEpurple, MEtan, MEblack, and MEblue were significantly different (*p* < 0.05) (Figure 3E). The interesting modules in MDD occupied a larger number, MEblack, MEgreen, MEmagenta, ME purple, MEturquoise, MEpink, MEmidnightblue, MEsalmon, MEblue, MEyellow, MEbrown, MEtan, MElightcyan, and MEgrey can all be considered as related modules (Figure 3F). According to these interesting modules, 24 genes in CAD and 567 genes in MDD were identified as hub genes. We performed GO enrichment analysis to these genes and yield various pathways. After filtering pathways with gene counts more than two, 20 overlapped GO pathways shared between CAD and MDD were identified (Table.S2). These pathways were mainly associated with immune response, indicating that immune-related pathways were both relevant to the initiation of CAD and MDD.

## Discussion

In this bioinformatic analysis of public database, we identified 24 DEGS shared by MDD and CHD. Enrichment analysis performed by clusterProfiler and Metascape indicated that the shared genes are mainly associated with immune function, ribosome biogenesis, regulation of peptidase activity, mitochondrial translation, protein catabolic process, and voltage-gated channel activity. The functional interactions of shared genes were also demonstrated by means of PPI network analysis. In addition, three hub genes (MMP9, RETN, and S100A8) were identified. Among these three hub genes, MMP9 and S100A8 both belongs to IL-17 signalling pathway while RETN also belongs to immune-related pathway. WGCNA algorithm was also applied and results showed that CAD and MDD shared 20 GO pathways related to immune response. Therefore, it could be summarised that MDD and CHD are genetically associated in the mechanism of immune function and ribosome biogenesis.

The results of GO biological process and KEGG suggest that the shared DEGs were enriched in lymphocyte and leucocyte homoeostasis and apoptotic process, alpha-beta T cell receptor complex, T cell receptor binding, IL-17 signalling pathway, adaptive immune system, and leucocyte differentiation. These findings are consistent with previous work reporting that immune systems and inflammation pathways are essential to the pathogenesis of both MDD and CHD. Many studies have shown that immune activation plays a pivotal role in MDD, demonstrated by T cell activation, and dysregulation (Maes, 2011, Maes *et al*., 2012, Osborne *et al*., 2020) and increased level of inflammatory cytokines, including pro-inflammatory cytokines, acute phase protein (Maes *et al*., 1992), C-reactive protein (CRP), interleukin (IL-1β), interleukin-6 (IL-6), tumour necrosis factor alpha (TNF-α), and interferon gamma (IFN-γ) (Maes *et al*., 1994). In addition, analyses of postmortem brain samples from suicide victims with MDD also found increased expression of innate immune genes and proteins. A study comprising 153 patients and 153 controls observed elevated levels of memory T helper cells and Th17 cells in patients at high risk of suicide (Schiweck *et al*., 2020), suggesting the premature ageing of the immune system. Moreover, increased concentration of inflammatory biomarkers at baseline is associated with poor prognosis in depressed patients (Haroon *et al*., 2018), while randomised controlled trial showed that the administration of anti-inflammatory treatment had special efficacy in treatment-resistant patients with high baseline inflammatory biomarkers (Raison *et al*., 2013). Specifically, the increased activities of inflammation in patients with MDD may affect endothelial functions and facilitate plaque formation, leading to disorders in cardiovascular system, and ultimately, CAD. It is widely established that atherosclerosis is a chronic inflammatory disease, demonstrated by the inflammatory activity of cholesterol and other molecular mechanisms. Emerging evidence suggests that circulating levels of acute phase protein, CRP, and IL-6 predict future cardiovascular events (Stewart *et al*., 2009) and CRP has already been used widely as a biomarker for cardiovascular risk. On the other hand, the dysfunction of inflammation in CAD may have influence on hypothalamic–pituitary–adrenal axis and the depletion of neurotrophic factors, destroying biological processes which play neuroprotective role against depression (Makhija and Karunakaran, 2013). Recently, a three-year follow-up study (Sforzini *et al*., 2019) found that higher levels of baseline high-sensitivity C-reactive protein (hsCRP) in CHD patients predict future development of depression and supported the inflammatory connection between CHD and MDD.

Our findings are consistent with other work suggesting that the ribosome-related pathways may account for the aetiology of both MDD and CHD. The eukaryotic ribosome is a ribonucleoprotein complex built of small 40S and large 60S subunit. In mammals, the subunits are composed of 28S rRNA, 5.8S rRNA, 5S rRNA, and various ribosomal proteins (Ghosh and Shcherbik, 2020). Ribosomes translate the information contained in mRNAs into functional proteins and are critical organelles for protein synthesis. Moreover, ribosomes are essential to neuron development and the activation of protein synthesis conducted by ribosomes is indispensable to synaptic plasticity. TJ Younts et al. found that the presynaptic protein synthesis by ribosomes played a crucial role in maintaining the long-term plasticity of GABA (neurotransmitter gamma-aminobutyric acid) release (Younts *et al*., 2016). In MDD, experimental evidence indicates that ribosomal genes were upregulated in the hypothalamus (Smagin *et al*., 2016) but were downregulated in hippocampus (Smagin *et al*., 2016, Zhang *et al*., 2019) in animal models, suggesting dysfunction of ribosomal gene expression is associated with the pathology of MDD. Hiroaki Hori et al. (Hori *et al*., 2018) found that increased expression levels of ribosomal genes were associated with stress vulnerability and were maximally upregulated in MDD patients. Furthermore, compared with healthy controls, the RNA sequencing-based genome-wide expression study of Darby and his colleagues (Darby *et al*., 2016) manifested the overexpression of ribosomal genes and the enrichment of ribosome pathway in the orbitofrontal cortex of MDD patients’ postmortem brains. However, the mechanisms underlying the association between ribosomal related pathways and MDD is unclear. In CHD, despite paucity of direct evidence of ribosome malfunction, Martinet and co-authors (Martinet *et al*., 2004) found that compared with the good-quality RNA isolated from negative samples, eleven out of twenty total RNA extracted from atherosclerotic specimens showed extensive fragmentation of 18S and 28S rRNAs and modification with the oxidative stress marker 8-oxoguanosine (8-oxoG), implying the possibility that the degradation of oxidised ribosomes negatively affect the survival and proliferation of smooth muscle and endothelial cell (Ghosh and Shcherbik, 2020). Yang Cao et al. found that γ2-AMPK suppress pre-rRNA transcription and ribosome biogenesis during cardiac stress and protect against ischemia/reperfusion injury in mouse model (Cao *et al*., 2017).

Interestingly, we also found that rhythmic process is associated with two diseases. The circadian rhythm abnormalities are well established in MDD patients. Symptoms in people with depression often show a diurnal pattern, presenting with mild symptoms in the morning lows and worsened in the evening. Such dysfunction often restores to normal after recovery. Circadian rhythm dysfunction also includes body temperature rhythms and hormone rhythms in the form of reduced amplitude in melatonin and disrupted cortisol secretion (Walker *et al*., 2020). Meanwhile, agomelatine, a melatonergic antidepressant, appeared to be effective in treating major depression. In individuals with CAD, a lower 24-hour rest-activity rhythm amplitude is found (Moon *et al*., 2023). Another study reviewed the role of cortisol, which is correlated with an increased risk of cardiovascular events. They conclude that regulating the circadian rhythm of cortisol may provide a potential platform for preventing cardiovascular events (Mohd *et al*., 2021). Therefore, we can infer that depression and CHD may be linked through disturbances by hormone rhythms. Rhythm-regulating drugs may also help patients with CHD.

All three hub genes identified in this study have been reported to be associated with MDD and CAD. MMP9 is the most important zinc containing enzyme of MMP family (Hassanzadeh-Makoui et al.,2020). Several studies have demonstrated that MMP9 polymorphisms is associated with increased risk of CAD in different population (Morgan *et al*., 2003; Moradi *et al*., 2017; Hassanzadeh-Makoui *et al*., 2020). In MDD, MMP9 is reported to be increased in the development of stress model of depression (Alaiyed *et al*., 2020; Bijata *et al*., 2022). Furthermore, Lutgendorf et al. reported higher levels of MMP9 in patients with a higher number of negative life events over the last 6 months (Lutgendorf *et al*., 2008). S100A8 has been identified as playing a significant role in all stages of development of heart disease in both human beings and mice through overexpression in various cardiovascular cell types (Averill *et al*., 2012). The study by Gamboa-Sánchez et al. reported up-regulation of S100A8 in MDD patients than healthy controls but shows no significant difference between patients with and without antidepressant treatment (Gamboa-Sánchez *et al*., 2023), laying groundwork for the investigation of S100A8 as potential biomarkers in MDD. However, this study only included 8 healthy volunteers and a total of 21 MDD patients. Larger sample sizes are warranted in future studies. Several studies have reported that RETN–420C > G and + 299 *G* > A genotypes might be associated with higher risk of CAD (Tang *et al*., 2008; Hussain *et al*., 2011) and higher expression level of RETN predicts higher rates of all-cause mortality (Chou *et al*., 2022). There are few reports on the RETN gene in depression, and the only two articles (Zhang *et al*., 2020; Sun *et al*., 2021) obtained the association between RETN and MDD through bioinformatics methods. In general, all these key genes are related to immune inflammation, suggesting that MDD and CHD are may share immune and inflammation-related pathogenesis, especially the differentiation and homoeostasis pathways of various immune cells, which explains the common comorbidity of CHD and depression.

The results of our study have several methodological challenges and limitations. First, our study lacks experimental validation. In particular, previous studies have only demonstrated that gene RETN is associated with depression from a bioinformatics perspective, lacking laboratory evidence. Future studies investigating RETN as a potential biomarker for depression may be feasible. Moreover, this study is based on cross-sectional data to analyse the relationship between CAD and MDD. Establishing a prospective cohort can be helpful to further explore the pathogenesis of the co-morbidity of the two diseases. Second, there is currently no standard method for combined matrixes construction. The sva R package used in this research is just one of them. Comparation of hub genes yielded by multiple methods may do favour in better data mining. Third, during WGCNA application, lack of demographic information limited our analysis of the relationship between genes and traits.

## Conclusion

In conclusion, our study identified shared DEGs between MDD and CHD. These genes were mainly enriched in inflammation pathways and ribosome biogenesis. Both of these related pathways have been reported to play an important role in the pathogenesis of MDD and CHD. Further studies are expected to verify the effectiveness and accuracy of hub genes in identifying comorbidity of MDD and CAD.

## Supporting information

Shi et al. supplementary material Shi et al. supplementary material

## Supplementary material

The supplementary material for this article can be found at https://doi.org/10.1017/neu.2024.40.
