# Supplementary material for: The genetic association between major depressive disorder and coronary heart disease
Source: Acta Neuropsychiatr. 2025 Mar 21;37:e49. doi: 10.1017/neu.2024.40 (PMC13130332; doi:10.1017/neu.2024.40)
Supplement: Shi et al. supplementary material [file S0924270824000401sup001.docx]

Fig.S1 PCA plots of major depressive disorder (MDD) matrixes before and after elimination of batch effects


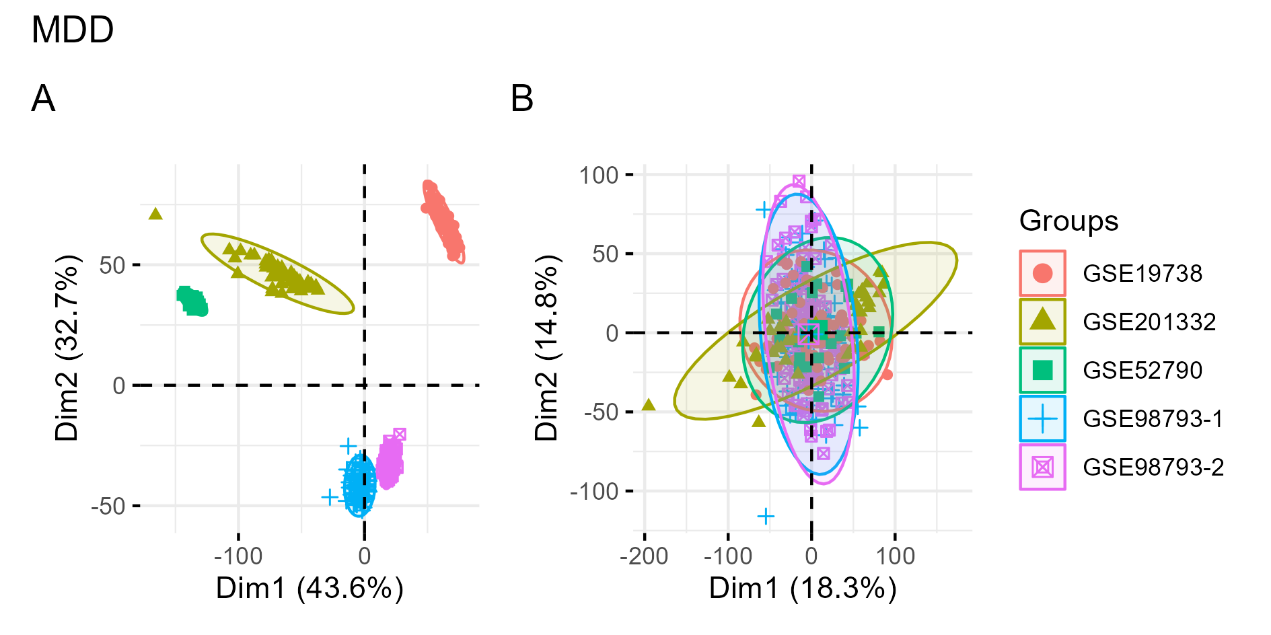


1. PCA plot of combined MDD matrix before eliminating batch effects. Data from different batches can be clearly shown to be divided into separate groups consistent with their sources.
2. PCA plot of combined MDD matrix after eliminating batch effects. The distance between data from different batches reduced.

Fig.S2 PCA plots of coronary heart disease (CAD) matrixes before and after elimination of batch effects


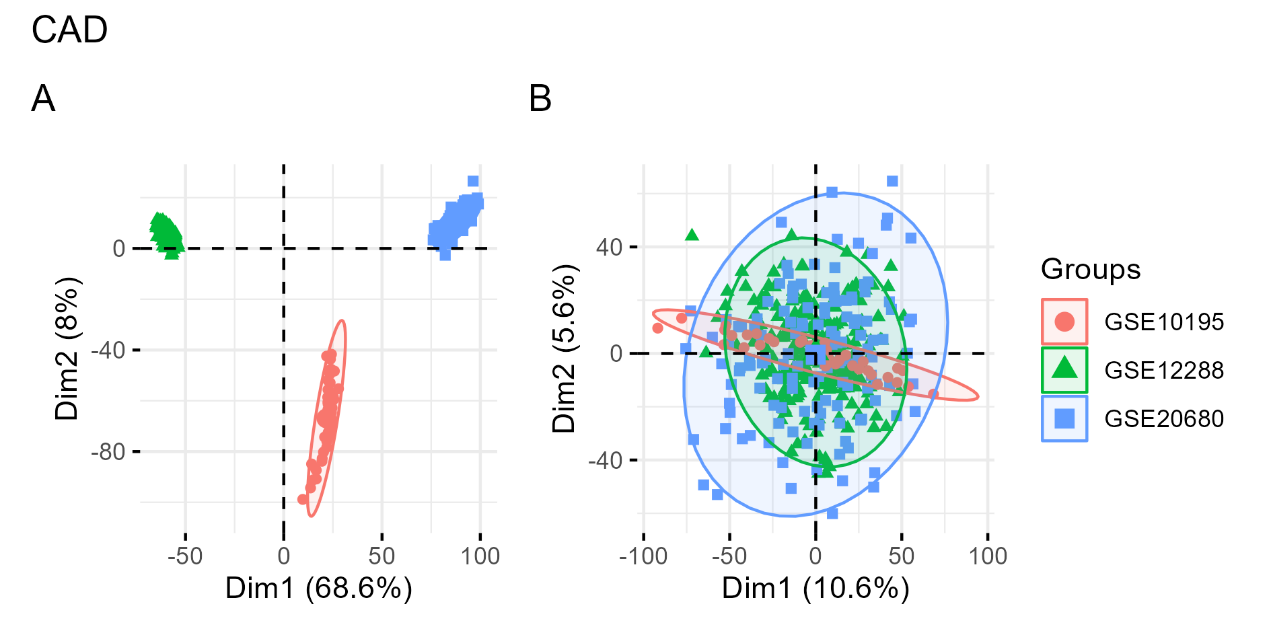


1. PCA plot of combined CAD matrix before eliminating batch effects. Data from different batches can be clearly shown to be divided into separate groups consistent with their sources.
2. PCA plot of combined CAD matrix after eliminating batch effects. The distance between data from different batches reduced.

Fig.S3 Enrichment results of overlapping genes from Metascape


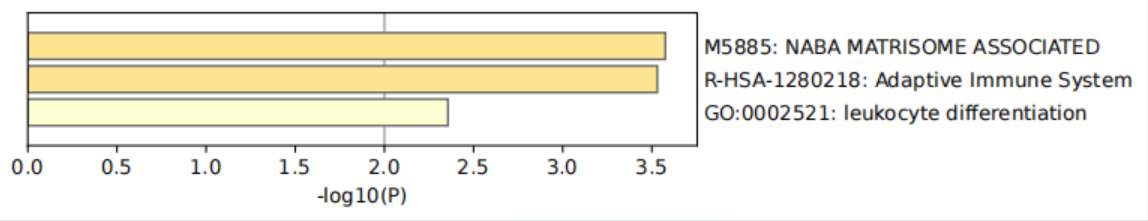


Fig.S4 MMP9 and S100A8 belong to the IL-17 signaling pathway


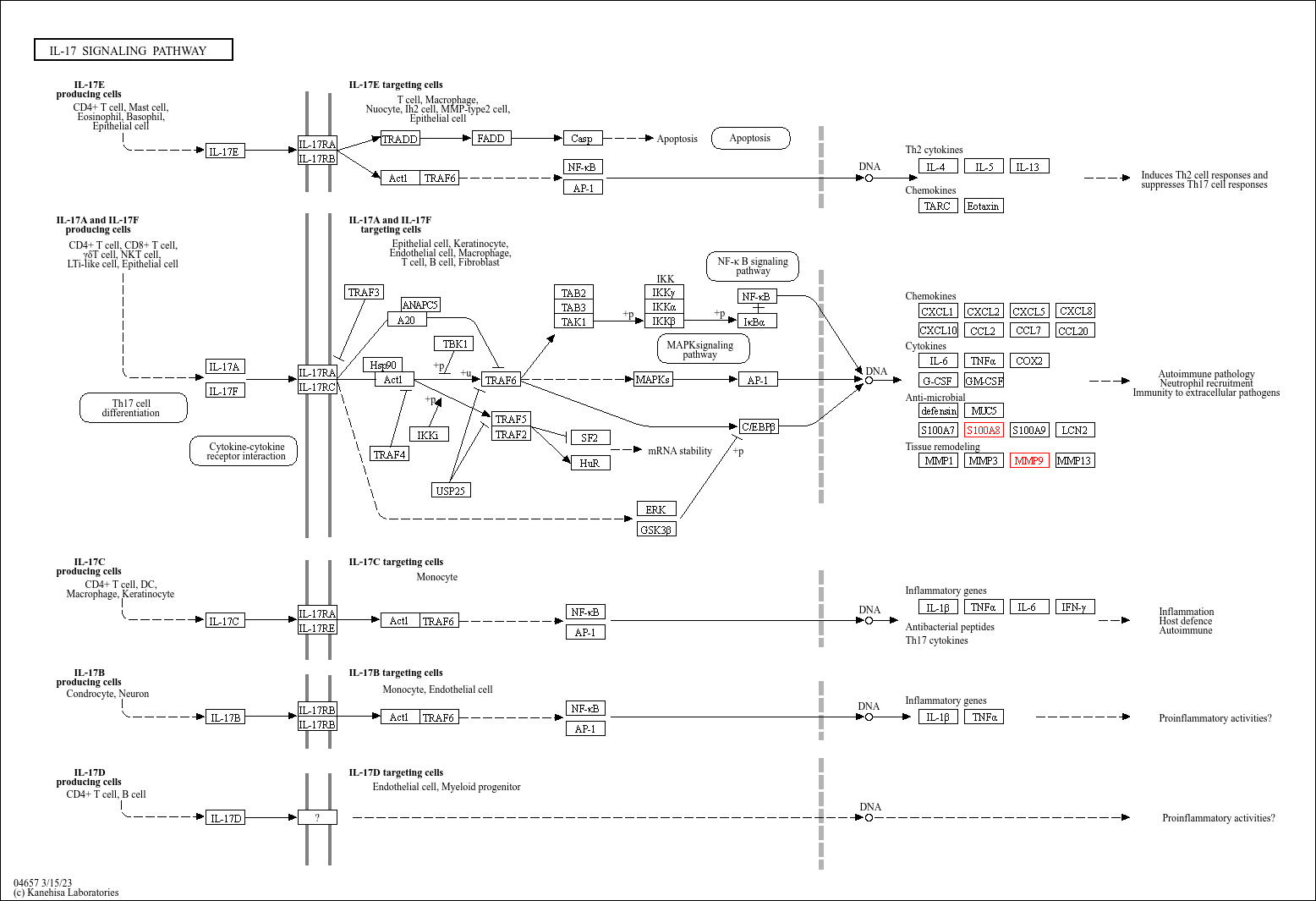


Fig.S5 Changes in IL-17 signaling pathway according to combined MDD matrix


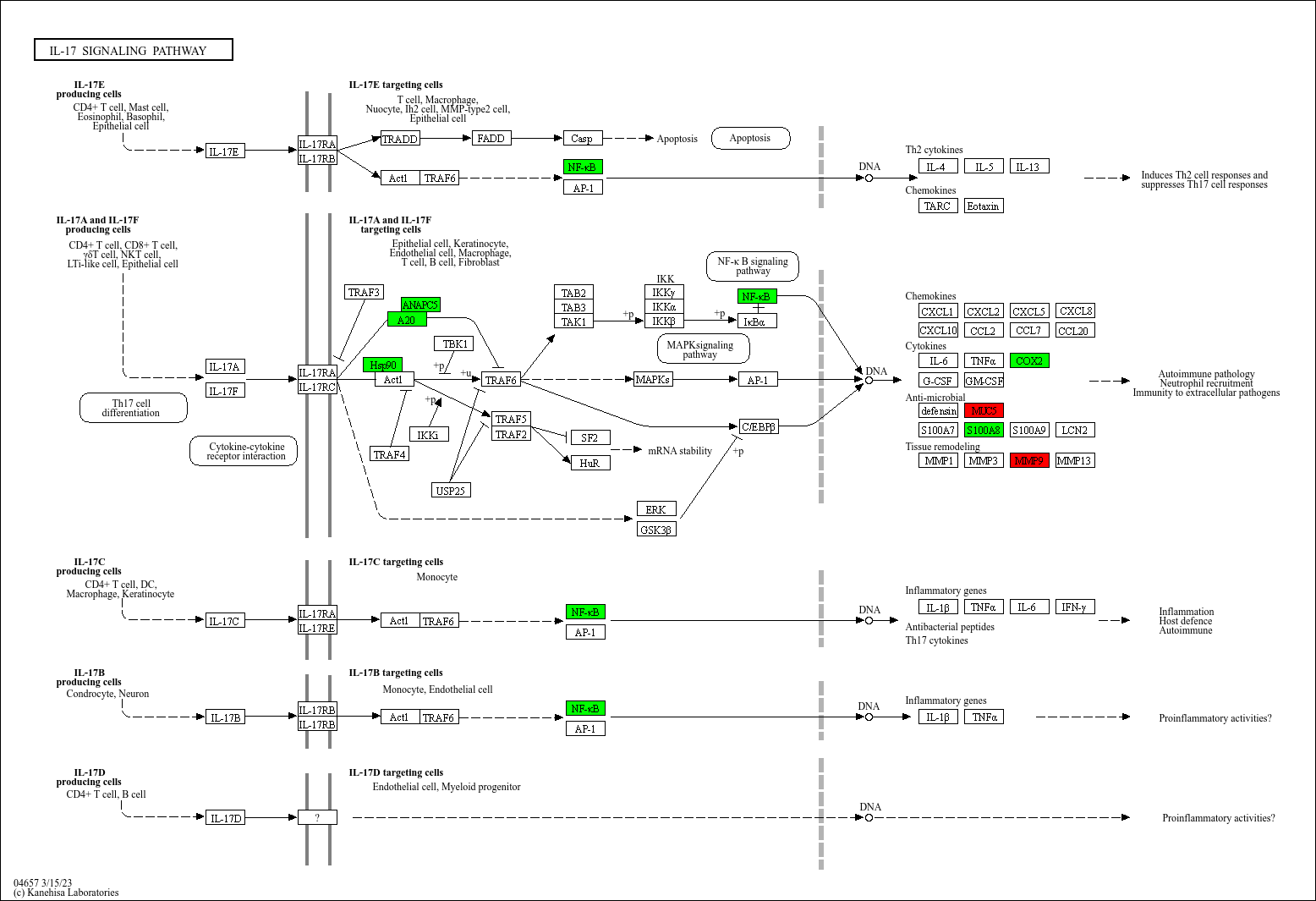


Fig.S6 Changes in IL-17 signaling pathway according to combined CAD matrix


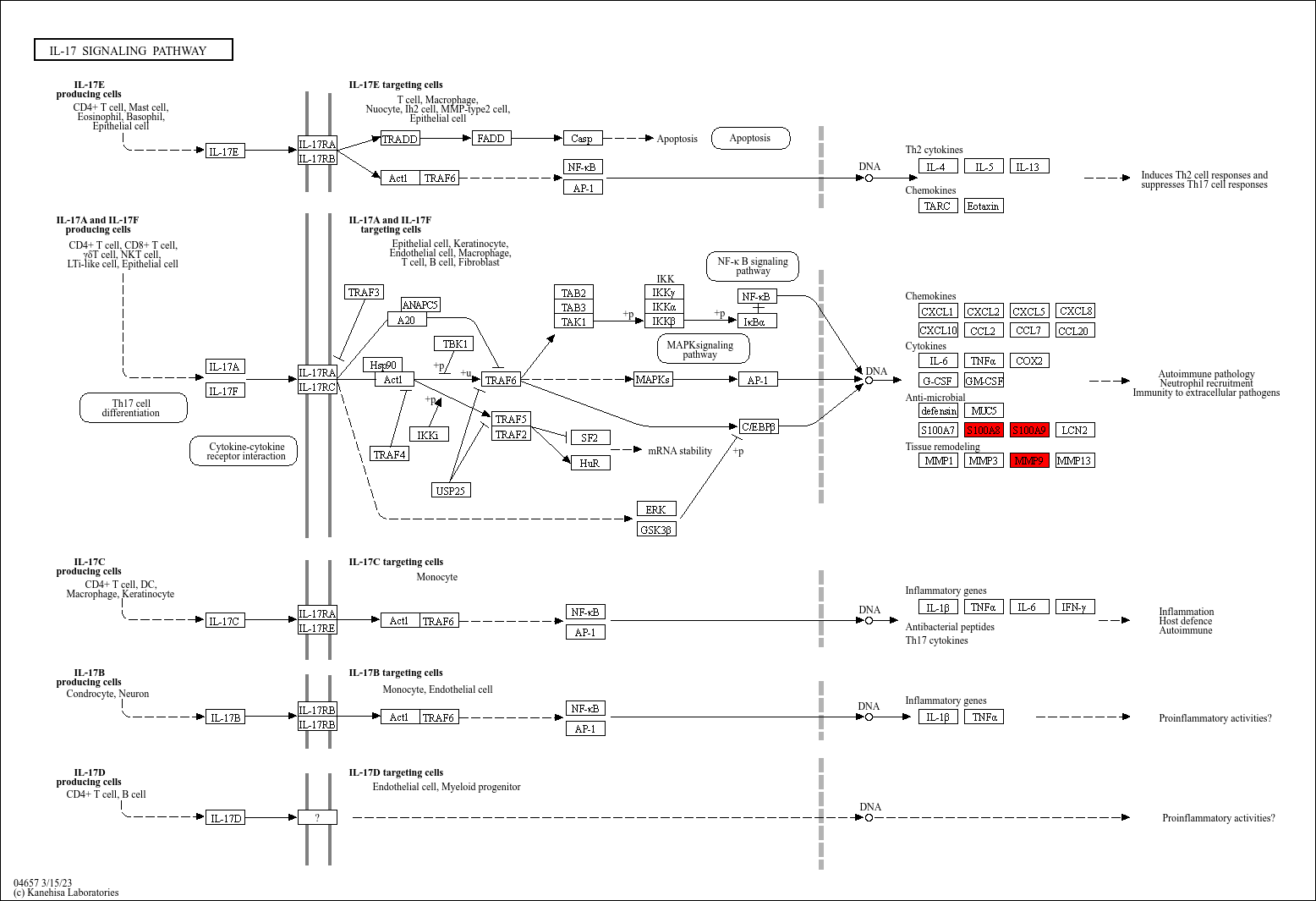


Table.S1 Results of CytoNCA

| **No.** | **Name** | **Subgragh** | **Degree** | **Eigenvector** | **Information** | **Betweenness** | **Closeness** |
| --- | --- | --- | --- | --- | --- | --- | --- |
| 1 | MMP9 | 2.275631 | 4.0 | 0.5772178 | 2.1710527 | 8.0 | 0.1388889 |
| 2 | RETN | 2.2288294 | 4.0 | 0.49999994 | 2.1710527 | 6.0 | 0.1369863 |
| 3 | S100A8 | 2.2288294 | 4.0 | 0.5 | 2.1710527 | 6.0 | 0.1369863 |
| 4 | CSTA | 1.5910151 | 2.0 | 0.28880772 | 1.7553192 | 0.0 | 0.13157895 |
| 5 | KCNJ15 | 1.5910149 | 2.0 | 0.2888077 | 1.7553192 | 0.0 | 0.13157895 |
| 6 | MRPS18C | 1.5430806 | 2.0 | 0 | 1.7553192 | 0.0 | 0.1 |
| 7 | MRPL13 | 1.5430806 | 2.0 | 0 | 1.7553192 | 0.0 | 0.1 |
| 8 | HERC2 | 1.5430806 | 2.0 | 0 | 1.7553192 | 0.0 | 0.1 |
| 9 | DOCK10 | 1.5430806 | 2.0 | 0 | 1.7553192 | 0.0 | 0.1 |
| 10 | LGALS2 | 1.5430806 | 2.0 | 0 | 1.7553192 | 0.0 | 0.1 |
| 11 | CLC | 1.5430806 | 2.0 | 0 | 1.7553192 | 0.0 | 0.1 |

Table.S2 Intersected pathway of CAD and MDD

| **No.** | **Description** |
| --- | --- |
| 1 | response to virus |
| 2 | defense response to virus |
| 3 | defense response to symbiont |
| 4 | antiviral innate immune response |
| 5 | immune response-activating signaling pathway |
| 6 | immune response-regulating signaling pathway |
| 7 | viral process |
| 8 | innate immune response-activating signaling pathway |
| 9 | viral genome replication |
| 10 | activation of innate immune response |
| 11 | viral life cycle |
| 12 | positive regulation of innate immune response |
| 13 | positive regulation of response to biotic stimulus |
| 14 | regulation of innate immune response |
| 15 | pattern recognition receptor signaling pathway |
| 16 | positive regulation of defense response |
| 17 | mononuclear cell differentiation |
| 18 | cytosolic pattern recognition receptor signaling pathway |
| 19 | lymphocyte differentiation |
| 20 | intracellular receptor signaling pathway |
